# Poor Outcome and Mortality in Patients with Lower Lung-Dominant Sarcoidosis

**DOI:** 10.1155/2023/3624344

**Published:** 2023-04-15

**Authors:** Kazunobu Tachibana, Masanori Akira, Toru Arai, Chikatoshi Sugimoto, Seiji Hayashi, Yoshikazu Inoue

**Affiliations:** ^1^Department of Respiratory Medicine, National Hospital Organization Kinki-Chuo Chest Medical Center, Sakai City, Osaka, Japan; ^2^Clinical Research Center, National Hospital Organization Kinki-Chuo Chest Medical Center, Sakai City, Osaka, Japan; ^3^Department of Radiology, Katano Hospital, Katano City, Osaka, Japan; ^4^Sugimoto Naika Clinic, Sakai City, Osaka, Japan; ^5^Department of Internal Medicine, Aihara Daini Hospital, Osaka City, Osaka, Japan

## Abstract

**Background:**

Pulmonary sarcoidosis predominantly affects the upper lung zones but sometimes affects the lower lung zones. We hypothesised that patients with lower lung zone-dominant sarcoidosis had lower baseline forced vital capacity, progressive restrictive lung function decline, and higher long-term mortality.

**Methods:**

We retrospectively reviewed clinical data including the pulmonary function tests of 108 consecutive patients with pulmonary sarcoidosis pathologically confirmed by lung and/or mediastinal lymph node biopsy from 2004 to 2014 from our database.

**Results:**

Eleven patients (10.2%) with lower lung zone-dominant sarcoidosis were compared with 97 patients with nonlower lung zone-dominant sarcoidosis. The median age of the patients with lower dominance was significantly older (71 vs. 56, *p* = 0.0005). The patient with lower dominance had a significantly lower baseline percent forced vital capacity (FVC) (96.0% vs. 103%, *p* = 0.022). The annual change in FVC was −112 mL in those with lower dominance vs. 0 mL in nonlower dominance (*p* = 0.0033). Fatal acute deterioration was observed in three patients (27%) in the lower dominant group. Overall survival in the lower dominant group was significantly worse.

**Conclusions:**

Patients with lower lung zone-dominant sarcoidosis had an older age and lower baseline FVC with disease progression and acute deterioration associated with higher long-term mortality.

## 1. Introduction

Sarcoidosis is a systemic granulomatous disease of unknown cause that occurs in pulmonary sites in 90% of cases [1]. Pulmonary sarcoidosis usually affects the upper lung zones [2, 3]; however, the lesions are sometimes distributed predominantly in the lower lung zones [2]. The course of pulmonary sarcoidosis has been widely studied using clinical and pulmonary function follow-up [4, 5], but, to our knowledge, no specific study has been reported of sarcoidosis with lower lung zone dominance. In a previous report, we presented a patient of sarcoidosis with lower lung field-dominant reticular shadows, who died from acute deterioration [6]. Although the autopsy findings suggested that the sarcoidosis was complicated by usual interstitial pneumonia, we were not able to determine whether sarcoidosis was complicated by interstitial pneumonia while the patient was alive. Therefore, in a clinical setting, we have no choice but to treat lower lung zone-dominant sarcoidosis as a distinctive phenotype. We hypothesised that patients with lower lung zone-dominant sarcoidosis led to decline in lung function with higher long-term mortality related to respiratory causes including acute deterioration episodes.

## 2. Subjects and Methods

### 2.1. Subjects

The study analysed patients diagnosed with pulmonary sarcoidosis in the National Hospital Organization Kinki-Chuo Chest Medical Center between April 2004 and March 2014. The diagnosis of sarcoidosis was based on a multidisciplinary diagnosis: consistent clinical features and bronchoalveolar lavage fluid analysis and confirmation of the presence of noncaseating epithelioid cell granulomas by lung and/or mediastinal lymph node biopsy, according to the WASOG (World Association of Sarcoidosis and Other Granulomatous Disorders) guidelines [7]. This study was approved by the Ethics Committee of the National Hospital Organization, Kinki-Chuo Chest Medical Center (approved on July 27, 2015; approval number 501).

### 2.2. Radiographic Assessments

CT scanners (HiSpeed Advantage and Light-Speed 16; GE Healthcare, Milwaukee, WI, USA) were used for the present study. Thin-section CT examinations were performed at 120 kVp and 160 mAs, with 1.5 mm collimation at 15 mm intervals. Lung zone dominance was classified into lower dominance and nonlower dominance by an expert chest physician (K. T.) and an expert chest radiologist (M. A.) without reference to the clinical or pulmonary function test results. They separately checked the images and discussed those cases where there was disagreement, approving the final radiographic assessment by consensus. Pulmonary sarcoidosis was classified as lower dominance in chest radiography when the total extent of pulmonary lesions was predominant in the lower lung, with the lower lung being defined as below the level of the inferior pulmonary veins [8]. The following CT patterns were recognised [9, 10]: (1) nodules (defined as those with diameters <1 cm), which included centrilobular nodules, subpleural nodules, and nodules along bronchovascular bundle; (2) conglomeration, which was defined as a large opacity ≥3 cm in diameter that often surrounded and encompassed the bronchi and vessels; (3) thickening of bronchovascular bundle; (4) interlobular septal thickening; (5) air-space consolidation, which was defined as an area of increased attenuation with obscuring of the adjacent bronchial walls and vessels; (6) ground-glass opacity, defined as an area of slightly increased attenuation in which the bronchial walls and vessels remained visible; (7) traction bronchiectasis, defined as irregular bronchial dilatation within areas of parenchymal abnormality; and (8) honeycombing, defined as an accumulation of cystic spaces with thickened walls. Pulmonary sarcoidosis with traction bronchiectasis, honeycombing, and cystic changes was defined as fibrotic sarcoidosis.

### 2.3. Measurements of Clinical Parameters

Pulmonary function tests, including forced vital capacity (FVC) and diffusing capacity of carbon monoxide (DLco), were performed using a CHESTAC-8800 (Chest M. I., Inc., Tokyo, Japan), according to the method described in the ATS 1995 update [11]. The values for forced vital capacity (FVC), forced expiratory volume in 1 s (FEV1), and DLco were related to % predicted values [12]. Serum angiotensin converting enzyme (ACE) (normal range, 8.3–21.4 U/L) was measured by modified colorimetric assay using Kasahara's method [13]. Serum Krebs von den Lungen-6 (KL-6) (normal range <500 U/L) and surfactant protein-D (SP-D) (normal range, <110 ng/mL) were measured by the enzyme-linked immunosorbent assay using commercially available kits (ED046; Eizai Co. Ltd., Tokyo, Japan, and SP-D ELISA; Yamasa Co., Tokyo, Japan; respectively) [14].

### 2.4. Assessment of Disease Progression

Multiple pulmonary function measurements for each of the patients were available for analysis, and the information from each subject was summarised by using the estimated yearly rate of change (slope), which was calculated from a linear regression of the pulmonary function data [15, 16].

### 2.5. Definition of Acute Deterioration

The following features based on previously used criteria for acute exacerbation in idiopathic pulmonary fibrosis [17, 18] were used to define an acute deterioration event: (1) three conditions were met including (i) progressive worsening dyspnea within one month, (ii) new ground-glass opacities or consolidation evident on HRCT, and (iii) a reduction in arterial oxygen tension (PaO_2_) at rest is of more than 10 Torr compared to previous measurements; (2) the exclusion of obvious causes of acutely impaired respiratory function, such as infection, pneumothorax, cancer, pulmonary embolism, or congestive cardiac failure.

### 2.6. Statistical Analysis

Differences in clinical data between the nonlower dominance and the lower dominance were compared using the Mann–Whitney *U* test for continuous variables and Fischer's exact test for categorical parameters. Cox proportional hazards regression models were used to calculate hazard ratios for mortality. Survival curves were based on the Kaplan–Meier method and compared using the log-rank test. Statistical analysis was performed with JMP Statistics Program, version 11 (SAS Institute Inc., NC, USA). For all statistical analyses, *p* < 0.05 was considered significant.

## 3. Results

### 3.1. Baseline Clinical Data of the Patients

**Figure 1:**
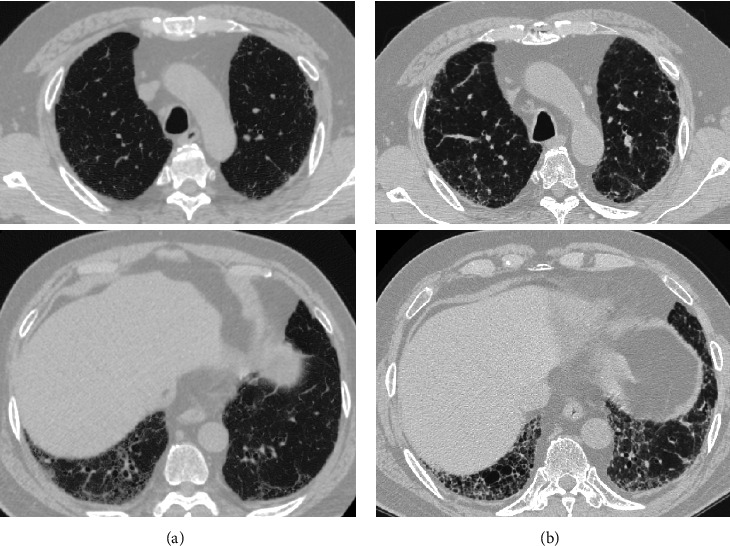
High-resolution CT scans of a representative 62-year-old man sarcoid patient at the time of diagnosis (a) and 3 years after diagnosis (b).

Among 139 patients identified in the database, 31 were excluded (no granulomas were detected histologically in the lung: *n* = 14; incomplete medical record: *n* = 4; no bronchoscopic study: *n* = 1; CT not available: *n* = 1; cutaneous sarcoidosis; *n* = 1; and incomplete data in our hospital due to being diagnosed initially in other institutes: *n* = 10). The remaining 108 patients were analysed, of whom 11 had lower dominance and 97 had nonlower dominance. At baseline, no significant differences were observed in the male/female ratio or serum angiotensin-converting enzyme (ACE), whereas the lower dominant group had a significantly older age (71 vs. 56, *p* < 0.001), higher serum Krebs von den Lungen (KL-6) levels (1046 U/mL vs. 342 U/mL, *p* < 0.001), higher surfactant protein D (SP-D) levels (193 ng/mL vs. 53.4 ng/mL, *p* < 0.001), and lower baseline percent forced vital capacity (FVC) (96.0% vs. 103%, *p*=0.022) (Table 1).  Table 2 shows the clinical characteristics of patients with lower lung zone-dominant sarcoidosis. Representative chest X-ray and HRCT scans of a patient with lower lung zone-dominant sarcoidosis are shown in Figure 1. Existence of diffuse pulmonary nodules and noncaseating granulomas raises the possibility that the patients with lower lung zone-dominant sarcoidosis might have chronic hypersensitivity pneumonitis. The patients with lower lung zone-dominant sarcoidosis had extrapulmonary sarcoid lesions, high levels of serum ACE, noncaseating granulomas in mediastinal lymph nodes, or perilymphatic nodular pattern: nodules along the bronchovascular interstitium, interlobular septa, and subpleural lung regions (Table 2). These findings therefore did not support the likelihood of chronic hypersensitivity pneumonitis. Six of 11 patients with the lower dominance had high serum B-type natriuretic peptide (BNP) or *N*-terminal-pro-BNP (NT-pro BNP) levels and had suspected pulmonary hypertension from their echocardiographic assessments. One of 97 patients with the nonlower dominance had high serum BNP levels and had suspected pulmonary hypertension from their echocardiographic assessment.

### 3.2. Radiographic and Histological Findings

Interlobular septal thickening, ground glass opacities, and traction bronchiectasis were significantly more prevalent in the lower dominant group (64%, 82%, and 64%, respectively) than in the nonlower dominant group (6%, 22%, and 2%, respectively) (*p* < 0.001) (Table 3). In the lower dominant group, noncaseating epithelioid cell granulomas were detected in transbronchial lung biopsy of the upper lobes in 10 of 11 patients and the lower lobes in 6 of 11 patients. Five patients had fibrosing mural alveolitis in the lower-lung zones (Table 2). In the nonlower dominant group, noncaseating epithelioid cell granulomas were detected in transbronchial lung biopsies of the upper lobes in 41 of 97 patients, the lower lobes in 19, and lobes that were not otherwise specified in 37; no patients had fibrosing mural alveolitis.

### 3.3. Rates of Change in Lung Function Tests as a Measure of Disease Progressions

**Figure 2:**
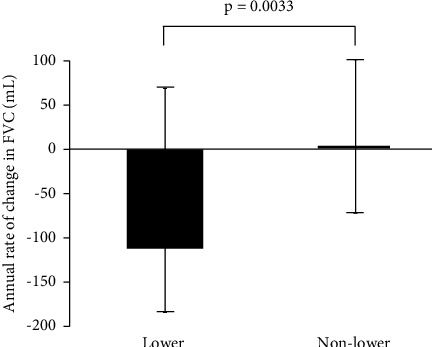
The annual rate of decline over time in forced vital capacity (FVC) in lower dominance and nonlower dominance.

The median number of tests per patient was 4 (range 2–6) for those with lower dominance vs. 4 (range 2–9) for those with nonlower dominance. After a median follow-up time of 3.2 years (range 1.0–5.9 years) for the lower dominant group and 5.0 years (range 1.0–9.0 years) for the nonlower dominant group, the annual rate of change in FVC was −112 mL (interquartile range (IQR): −184–69 mL) and 0.00 mL (IQR: −40–74 mL), respectively (*p*=0.0033) (Figure 2). The annual rate of change in DLco was 0.000 mL/min/mmHg (IQR: −1.244–0.677 mL/min/mmHg) with the lower dominant group vs. −0.081 mL/min/mmHg (IQR: −0.769–0.245 mL/min/mmHg) with the nonlower dominant group (*p*=0.50).

### 3.4. Outcome and Mortality

**Figure 3:**
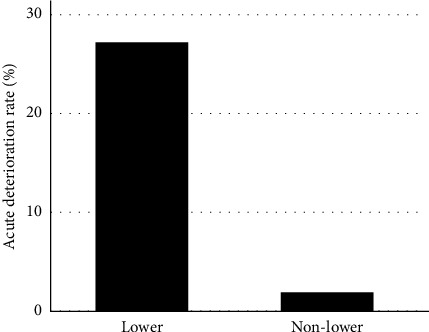
Comparison of the acute deterioration rate between patients with lower dominance and nonlower dominance.

**Figure 4:**
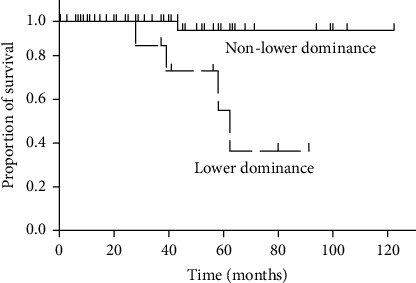
Comparison of overall survival between patients with lower dominance and nonlower dominance. Survival of patients was calculated from the date of first evaluation with pulmonary sarcoidosis. The lower dominant group (all: dashed line and except for the atypical cases: dotted lines) showed significantly worse survival rates than the nonlower dominant group (the solid line). *p* < 0.001 in the log-rank test. +: censored patients.

The median observation period of the lower dominant group, the nonlower dominant group, and both groups combined was 41 months (range 12–91 months), 58 months (range 12–140 months), and 58 months (range 12–140 months), respectively. Of the lower-dominant patients, five (46%) were given corticosteroids and two (18%) were given immunosuppressants (one patient was given azathioprine, and the other was given cyclosporine) during the course of the disease. The remaining six patients were given no treatment because three patients had pulmonary infections and the remaining three patients were stable. In contrast, in the nonlower dominant group, 4% of patients were given corticosteroids. Acute deterioration episodes were observed in three patients (27%) in the lower dominant group but in only one patient (1%) in the nonlower dominant group (Figure 3). The lower dominant group showed significantly worse overall survival with median survival time of 60 months (*p* < 0.001) (Figure 4). Mortality was caused by acute deterioration episodes (three patients) and chronic respiratory failure (one patient) in the lower dominant group and by lung cancer (one patient) in the nonlower dominant group. Annual FVC change was not significantly associated with overall survival (hazard ratio = 0.0301 and *p*=0.0818).

### 3.5. Comparing Fibrotic Sarcoidosis in the Lower Dominant Group with Fibrotic Sarcoidosis in the Nonlower Dominant Group

Patients with fibrotic sarcoidosis in the lower dominant group (*n* = 7) were compared with those in the nonlower group (*n* = 3). After a median follow-up time of 3.4 years (range 3.1–6.7 years) for patients with fibrotic sarcoidosis in the lower dominant group and 9.0 years (range 1.0–9.0 years) in the nonlower dominant group, the annual rate of change in FVC was −206 mL (IQR: −390–−106 mL) and −34.0 mL (IQR: −62–−15 mL), respectively. Four of the seven patients died in the lower-dominant group, and none of the three patients died in the nonlower dominant group.

## 4. Discussion

The main results of the present study were as follows: (1) patients with lower lung zone-dominant sarcoidosis had lower baseline forced vital capacity with progressive decline in restrictive lung function decline; (2) patients with lower lung zone-dominant sarcoidosis had a significantly decreased survival as compared with the nonlower lung zone-dominant group; and (3) mortality was mainly related to acute deterioration episodes.

The lower lung dominance had significantly higher serum KL-6 and SP-D levels compared with the nonlower lung zone-dominant group. Serum KL-6 and/or SP-D can be elevated in patients with pulmonary granulomatous diseases including sarcoidosis and berylliosis [19–21]. Meanwhile, serum levels of KL-6 reflect fibrotic processes and levels of SP-D are elevated in patients with idiopathic pulmonary fibrosis [22, 23]. In the lower lung zone-dominant group, interlobular septal thickening, ground glass opacities, and traction bronchiectasis, which are putative features of fibrotic interstitial pneumonias [10], were more prevalent. Moreover, five patients in the lower lung zone-dominant group had fibrosing alveolitis. Combined, the histopathology of lower lung dominance might include fibrosing interstitial pneumonia or lung injury.

We have presented patients with lower lung zone-dominant sarcoidosis with fibrotic signs, including traction bronchiectasis in HRCT; most of them are old in age and male. In general, fibrotic interstitial lung diseases (ILDs) also involve lower lungs in elderly male patients and have poor prognosis and high mortality [24]. We reported a case of lower lung zone-dominant sarcoidosis, in which sarcoidosis was complicated by usual interstitial pneumonia [6]. This determination was based on the fact that the autopsy specimens showed diffuse alveolar damage and no concentric fibrosis or no concentric lamellar calcifications (Schumann bodies), which are characteristics of the fibrotic stage of sarcoidosis [25, 26]. In contrast, Nobata et al. reported a case of lower lung zone-dominant sarcoidosis, in which they noted the possibility that sarcoidosis was at the fibrotic stage of sarcoidosis because the fibrosis was distributed along the bronchovascular bundles on surgical lung biopsy [3]. Only one autopsy specimen from the lower dominance was available in the present study. Further studies with larger numbers of the lower-dominant group diagnosed on surgical lung biopsy and/or autopsy are needed to determine whether sarcoidosis and another fibrotic ILD overlapped or whether the fibrosis was attributed to pulmonary sarcoidosis itself.

We showed that annual FVC change, a putative marker of disease progression, was not significantly associated with mortality, probably due to fatal acute deterioration in the lower dominant group. In progressive fibrosing interstitial lung disease, relative FVC decline ≧10% in the previous 24 months was shown to be associated with mortality [15]. FVC has been used to define disease progression in recent trials on patients with progressive pulmonary fibrosis [27, 28]. We also showed that the baseline FVC in the lower dominant group was significantly lower, and despite treatment with steroids and immunosuppressants, the yearly decline in FVC was more marked than that observed for the nonlower group. In addition, similar results were reported in sarcoid patients with pulmonary fibrosis, that is, stage IV sarcoidosis [29]. Moreover, we showed that the lower dominant group had higher mortality. We also showed that patients with fibrotic sarcoidosis in the lower dominant group had relatively higher mortality compared with those in the nonlower group, although the number of cases in both groups is limited and statistical analysis was not performed. Thus, when lower lung-dominance is seen in sarcoid patients, it necessitates careful follow-up with serial pulmonary function tests.

Recently completed randomised controlled trials have demonstrated the clinical efficacy of nintedanib, an antifibrotic agent, in patients with progressive fibrosing interstitial lung disease (PF-ILD) [28]. Moreover, in progressive pulmonary fibrosis, a conditional recommendation was made for nintedanib in the ATS/ERS/JRS/ALAT guideline [24]. The use of antifibrotic agents might be warranted to slow down the decline in FVC and to reduce the mortality in the future.

The present study has some limitations. First, this is a single-centre retrospective study. Despite this, we had access to all available consecutive data to maximised accurate diagnosis of pulmonary sarcoidosis with a distinct clinicopathological entity. In addition, the survival of our cohort was similar to that of other reports based on large national cohorts [5, 29, 30]. Furthermore, we were able to follow individual subjects longitudinally, affording us the opportunity to detect acute deterioration of pulmonary sarcoidosis. The second limitation is including a limited number of patients with the lower-dominance. Nonetheless, we showed significant statistical differences in the annual rate of change in FVC as a disease progression measure and overall survival between the two groups. Further studies with larger numbers of the lower-dominant group are needed to confirm these results. Third, not all patients in the lower-dominant group had surgical lung biopsy-confirmed fibrosing interstitial pneumonia in the lower-lung zones. However, transbronchial lung biopsy revealed that 5 out of 11 patients had fibrosing alveolitis in the lower-lung zones, raising the possibility that histologically, a significant number of patients in the lower-dominant group had fibrosing interstitial pneumonia. Finally, right heart catheterization for assessing pulmonary hypertension, which is sometimes observed in advanced pulmonary sarcoidosis or interstitial pneumonia [31–34], was not performed in our cohort. However, this would not affect the decline in FVC and the mortality because pulmonary hypertension could reduce only the DLco value and because no patients died of right-sided cardiac failure.

## 5. Conclusions

Patients with lower lung zone-dominant sarcoidosis had an older age and lower baseline FVC with disease progression and acute deterioration associated with higher long-term mortality related to respiratory causes, particularly acute deterioration. Further studies are warranted to determine whether sarcoidosis and another fibrotic ILD overlapped or whether the fibrosis was caused by pulmonary sarcoidosis itself.

## Figures and Tables

**Table 1:** Baseline characteristics in patients with lower and nonlower lung-dominant sarcoidosis.

	Lower dominance *n* = 11	Nonlower dominance *n* = 97	*p* value
Age	71 (62–73)	56 (36–65)	0.0005
Male/female	8/3 (73%)	34/63 (35%)	0.0989
Smoking history			
CS/Ex/never	0/7/4	23/22/52	0.3480
Extrapulmonary lesion, yes	6 (55%)	62 (64%)	0.5315
Treatment			
Steroids/immunosuppressants	5/2	4/0	—
ACE (U/L)	22.4 (16.9–37.2)	20.6 (15.2–25.7)	0.0839
KL-6 (U/mL)	1046 (699–2495)	342 (232–447)	<0.0001
SP-D (ng/mL)	193 (144–295)	53.4 (31.5–80.0)	<0.0001
Ly% (BALF)	29.4 (15.2–52.1)	29.4 (19.2–45.4)	0.6627
CD4/CD8 (BALF)	6.30 (4.48–8.16)	4.50 (2.65–7.55)	0.2914
%FVC	96.0 (63.5–108.7)	103 (90.0–121)	0.0220
%DLco	63.2 (44.1–89.6)	82.6 (69.6–93.5)	0.0505

Values are *n* (%) or median (interquartile range). CS: current smokers; Ex: ex-smokers; never: never-smokers; ACE: angiotensin converting enzyme; KL-6: Krebs von Lungen-6; SP-D: surfactant protein-D, BALF: bronchoalveolar lavage fluid; FVC: forced vital capacity; DLco: diffusing capacity of carbon monoxide.

**Table 2:** Clinical characteristics in patients with lower lung-dominant sarcoidosis.

Patient no.	Age (years), gender	Smoking status	Perilymphatic nodules	Diagnostic procedure	Histology	Extrapulmonary lesions	ACE (U/L)	Treatment	Outcome
1^*∗*^	62, male	Ex	+	TBLB, Med	Gr, F	Skin	25.1	PSL, CsA	Died (AD)
2	60, male	Ex	+	TBLB	Gr	Eye, PG	28.7	PSL	Alive
3	70, female	Never	+	TBLB, Med	Gr, F	Eye	20.4		Alive
4	70, male	Ex	−	TBLB	Gr	—	39.3		Alive
5	72, male	Ex	−	TBLB, SL	Gr	—	20.2	PSL	Died (AD)
6	73, male	Ex	+	TBLB	Gr, F	—	11.2		Died
7	71, female	Never	+	TBLB	Gr, F	Eye	22.4		Alive
8	71, female	Never	+	TBLB	Gr	—	47.0		Alive
9	55, male	Ex	+	TBLB	Gr	—	16.9	PSL, AZP	Died (AD)
10	75, male	Ex	+	TBLB	Gr, F	Eye	15.7	PSL	Alive
11	75, male	Never	+	TBLB	Gr	Eye, skin	37.2		Alive

Ex: ex-smokers; never: never-smokers; TBLB: transbronchial lung biopsy; Med: mediastinoscopy; SL: surgical subcarinal lymphnode biopsy; Gr: granulomas; F: fibrosing mural alveolitis, PG: parotid glands; PSL: prednisolone; AZP: azathioprine; CsA: cyclosporine; AD: acute deterioration; ^*∗*^ reference [6].

**Table 3:** Radiographic (high-resolution computed tomography) presentations in patients with pulmonary sarcoidosis.

	Lower dominance *n* = 11	Nonlower dominance *n* = 97	*p* value
Centrilobular nodules	4 (36%)	53 (51%)	0.343
Subpleural nodules	6 (55%)	40 (38%)	0.523
Nodules along bronchovascular bundle	2 (18%)	36 (33%)	0.321
Conglomeration	0 (0%)	2 (2%)	—
Thickening of bronchovascular bundle	1 (9%)	16 (15%)	1.000
Interlobular septal thickening	7 (64%)	6 (6%)	<0.0001
Air-space consolidation	2 (18%)	19 (18%)	1.000
Ground glass opacities	9 (82%)	23 (22%)	0.0002
Traction bronchiectasis	7 (64%)	3 (2%)	<0.0001
Honeycombing	0	0	—

## Data Availability

The data used to support the findings of this study can be obtained from the corresponding author upon reasonable request.
